# Organ Donation Motivators and Demotivators Among Families of Brain‐Dead Patients in Southeast Iran: A Qualitative Study

**DOI:** 10.1002/hsr2.73033

**Published:** 2026-08-12

**Authors:** Mehdi Ahamdinejad, Faezeh Ghasemi, Farideh Razban

**Affiliations:** ^1^ Neuroscience Research Center, Institute of Neuropharmacology Kerman University of Medical Sciences Kerman Iran; ^2^ Student Research Committee, Razi Faculty of Nursing and Midwifery Kerman University of Medical Sciences Kerman Iran; ^3^ Nursing Research Center Kerman University of Medical Sciences Kerman Iran

**Keywords:** brain death, Cadaveric organ donation, cultural barriers, motivators, organ Procurement, qualitative method

## Abstract

**Background:**

Cultural, social, and religious factors play an important role in the decision‐making of families of patients with brain death regarding organ donation.

**Objective:**

This study was conducted to identify the organ donation motivators and demotivators among the families of brain‐dead patients in Southeast Iran.

**Method:**

This qualitative study employed a conventional content analysis approach. Eleven family members of brain‐dead patients who had consented to organ donation participated in in‐depth, semi‐structured interviews.

**Results:**

A total of 241 codes, 19 sub‐subcategories, 8 subcategories, 2 primary categories, and one main category were identified. The main category derived was “Difficult dilemma” which included two primary categories: “Organ donation motivators” and “Organ donation demotivators” The primary category “Organ donation motivators” comprised subcategories such as “Discussing donation considerably,” “Patient's wishes awareness,” “Emotional/psychological support,” “Considering donation reasonable,” and “Considering donation valuable.” The primary category “Organ donation demotivators” consisted of subcategories such as “Discussing donation inconsiderately,” “Distrust in providers,” and “Considering donation unfavorably.” The findings indicate that the decision to donate organs is not solely an individual choice, but rather the outcome of an interplay between psychological and social factors. In this context, both motivating and demotivating influences interact and exert mutual effects.

**Conclusion:**

Based on the results of this study, it is suggested that healthcare professionals improve and professionalize their communication with families to encourage them to make informed decisions to donate organs of patients with brain death. Additionally, the role of the media in raising public awareness should be emphasized, religious leaders should take a more active role, and further studies should be conducted in this field.

## 1 Introduction

Organ donation offers a life‐saving solution for patients needing organ transplantation. However, many individuals, including those in Iran, face severe complications or death while waiting for available organs [1]. Brain‐dead patients are among the most important sources for procuring transplant organs and tissues [2]. Brain death indicates the irreversible loss of all brain functions, including activity in the brainstem and cerebrum [3]. The decision to donate the organs of brain‐dead individuals is made mainly by their family members [4].

The decision‐making process for donating the organs of brain‐dead individuals is very complex and difficult for their families. They have to make such a significant decision in a short period while dealing with the grief and sorrow of losing a loved one [5]. Many people do not communicate their wishes for organ donation after brain death to their families while they are healthy. As a result, families may oppose organ donation when the time comes [6]. In some cultures, discussing death is viewed as uncomfortable or taboo, which can create reluctance to talk about death and organ donation with family members [7]. At times, despite being aware that their patient's desire was to donate their organs, the difficulties and complexities surrounding this decision lead them to decline consent for organ donation.

Several qualitative studies outside of Iran examined the experiences of families in Canada [8] and India [9], as well as organ donation coordinators in India [10], regarding the barriers and facilitators to organ donation. These studies emphasized that better family support, improved communication with health care providers, and addressing family concerns can facilitate the donation process. A 2014 review [11] also highlighted these barriers and facilitators from the perspectives of families of brain‐dead patients.

Several qualitative studies [5, 6, 12, 13, 14, 15] have previously explored the experiences of families of brain death patients regarding organ donation decision making in Iran. In all these studies, religious and sociocultural factors played a significant role in families' decision‐making regarding organ donation. Some religious and sociocultural factors act as barriers, whereas others serve as facilitators. Other influencing factors in decision‐making include incorrect beliefs about the sale of loved ones' organs [13], the level of awareness about brain death and organ donation [6, 12], family interactions [6, 13, 14], and the performance of health care providers [5, 6, 12].

Therefore, cultural attitudes and beliefs should be considered among the most important influencing factors in the decision‐making process regarding organ donation [13]. In different cultures, attitudes toward death and organ donation vary. On the one hand, the types of relationships between individuals differ across families from various cultures. Thus, by conducting qualitative research and understanding different cultures and their perspectives on brain death and organ donation, as well as families' experiences with organ donation, a favorable environment can be created to encourage families to make informed decisions to donate organs. The literature review indicates that there have been no qualitative studies in Iran [5, 6, 12, 13, 14, 15] specifically designed to thoroughly investigate the motivators and demotivators of organ donation. Additionally, it is noteworthy that such studies have not been conducted in the southeastern region of Iran. Given the country's vastness—spanning approximately 1.648 million km^2^ with a population of around 90 million—and its cultural and ethnic diversity, the cultural dynamics of Iranians in the southeast differ significantly from those in other regions. Iran is characterized by a diverse array of Islamic groups, including both Shiite and Sunni sects, as well as various ethnic communities such as Baloch, Persian, Arab, and Turkish. The majority of Iranians, including those who participated in this study, identify as Shiite Muslims and are of Persian ethnicity. The first and third authors have several years of experience working in the ICU and have frequently encountered brain‐dead patients and the issue of organ donation. They were interested in conducting this study due to their concern for facilitating organ donation. Therefore, the present study was conducted to answer the question of what organ donation motivators and demotivators are among families of brain‐dead patients in Southeast Iran.

### 1.1 Context

Iran is a country located in the Middle East with a population of over 90 million [16]. Traffic accidents are the third leading cause of death in Iranians after cardiovascular events and stroke [16]. Young men who sustain head trauma in motor vehicle crashes constitute the largest group of brain death victims in Iran [1].

In Iran, by the end of 2019, there were 6240 patients on the waiting list for a kidney, 814 for a liver, and 768 for a heart transplant. The availability of organs for patients on the waiting list is insufficient to meet the growing demand [1]. In Iran, around 4000 brain‐dead individuals each year are eligible for organ donation, yet only about 25% of them actually proceed with the donation [15]. There are currently approximately 25,000 patients on the transplant waiting list in Iran, and 12 of these patients die every day because they do not receive a transplant [17]. The national deceased donation rate in Iran increased 19.06‐fold from 2002 to 2019, from 0.75 to 14.3 per million population (PMP) [1]. In other Islamic countries, including Malaysia and the Gulf nations (Bahrain, Kuwait, Oman, Qatar, United Arab Emirates, and Saudi Arabia), the rate of organ donation from deceased individuals remains significantly low, although there has been notable progress in recent decades [18, 19, 20].

The laws regarding organ and tissue donation in Iran were passed in 2000 after they were approved by religious authorities in the National Assembly [21]. According to existing laws in Iran, organ procurement from deceased individuals is conducted under the principle of informed consent (Opt‐In or explicit consent). According to this law, a person's organs can only be used if they have explicitly consented during their lifetime, such as by obtaining a donation card. In addition to the individual's consent, the family must also grant permission for the donation at the time of brain death, and possessing a donation card does not serve as legal authorization for donation [1, 21]. Some countries, such as the UK, implement an opt‐out policy to boost the number of organ donors. This policy presumes that all individuals wish to be organ donors after their death, and those who do not want to participate must explicitly state their wishes, typically by signing an official document to that effect [7].

According to the Research Center of Iran's Parliament [22], brain death is defined as the irreversible loss of all brain activity, including the cortex, subcortex, and brainstem. Confirmation involves a team of four physicians—a neurologist, neurosurgeon, internist, and anesthesiologist—who each assess the patient separately using tests like brainstem reflex evaluations and apnea testing. Consensus is required among all four to confirm brain death; if there is any disagreement, additional tests will be conducted. Those diagnosing brain death must not be part of the transplant teams.

Most Iranians (96.6%) are Muslim, and religious beliefs are the most important factor influencing their decision to donate organs [23, 24]. On the other hand, altruism and religious beliefs are the two main factors encouraging Iranians to donate organs [25].

## 2 Methods

### 2.1 Study Design

To explore the experiences of family members of brain‐dead patients regarding organ donation, qualitative content analysis was utilized. This method aligns with the objectives and research questions of the study. It systematically characterizes both explicit and implicit information within qualitative data, such as interview transcripts. It produces a structured representation of recurring themes and utilizes coding to identify and elucidate the range of factors present across different cases. For qualitative content analysis, three approaches—conventional, directed, and summative—are proposed. In the present study, the conventional content analysis method was applied via the approach of Graneheim and Lundman [26, 27].

### 2.2 Research Setting

This study was conducted at the Kerman University of Medical Sciences in southeastern Iran. The qualitative research environment is the real setting where phenomena occur, meaning the places where the participants reside and their experiences unfold. The interviews were conducted in locations where the participants felt comfortable and at ease, such as their homes.

### 2.3 Sampling, Participants and Data Collection

The participants in this study were families of organ donor patients with brain death. A purposeful sampling method was used to gather rich information. The inclusion criteria for the study included being over 18 years old, having experience with organ donation following the brain death of an immediate family, being willing to voluntarily participate in the current study, having an acceptable ability to communicate and express inner feelings and emotions, and understanding abstract concepts. The exclusion criterion included family withdrawal from the study.

Throughout the study, efforts were made to select participants who had rich experience regarding the phenomenon in question. A list of patients was prepared by visiting the Organ Transplant Unit of Kerman University of Medical Sciences, and their families were contacted. One family member was enrolled in the study for each brain‐dead patient. All of these families gave their consent to donate their patients' organs, despite some raising concerns. Maximum variation sampling was employed to access extensive and deep data. In total, interviews were conducted with 11 participants (one brother, two sisters, two fathers, three mothers, and three spouses of patients with brain death) (Table 1). Interviews were conducted nearly 3 months to 2 years following brain death and organ donation. The families were informed of the patient's brain death and the organ donation request by the liaison from the organ donation team. Due to time limitations, this notification occurred promptly after the brain death diagnosis. The families were provided with only a few days to make their decision.

**Table 1:** Participants' characteristics.

Code	Family member characteristics						Patients' characteristics		
	Relation to deceased	Gender	Age	Marital status	Education	Occupation	Age	gender	Diagnosis
P1	Brother	Male	41	Single	Diploma	Worker	20	male	Head trauma
P2	Mother	Female	50	Married	Diploma	Shopkeeper	24	male	SAH^a^
P3	Father	Male	43	Married	Diploma	Worker	11	female	CVST^b^
P4	Mother	Female	54	Married	Diploma	Housewife	19	male	Head trauma
P5	Wife	Female	37	Married	Secondary School	Housewife	39	Male	Head trauma
P6	Wife	Female	45	Married	Diploma	Housewife	47	Male	VTE^c^
P7	Sister	Female	30	Married	Bachelor's Degree	Housewife	26	female	Head trauma
P8	Father	Male	60	Married	Associate Degree	retired	28	female	Head trauma
P9	Sister	Female	35	Single	Bachelor's Degree	Teacher	20	male	Head trauma
P10	Wife	Female	44	Married	Diploma	Housewife	48	male	Massive stroke
P11	Mother	Female	47	Married	Diploma	Housewife	32	male	Head trauma

^a^ SubArachnoid hemorrhage.

^b^ Cerebral venous sinus thrombosis.

^c^ Venous thromboembolism embolism.

Three families were reluctant to participate in the study because they found it difficult to recall events related to the deaths of their loved ones. All participants were interviewed only once. In four interviews, a family member of the interviewee was present, but they did not interfere in the interview process.

The second and third authors, both female, conducted the interviews simultaneously and noted the participants' nonverbal reactions. To accurately capture the participants' experiences, the interviewers aimed to minimize their influence during the interviews. The second author was a medical student at the time of the study, while the third author was a faculty member and an assistant professor. During her PhD studies, she received training in qualitative research (e.g, conducting interviews), and her dissertation was a qualitative study. She has collaborated on several qualitative studies.

Before conducting the interviews, the researcher contacted the participants by phone to introduce themselves and explain the study's objectives and process. The researcher aimed to gain the participants' trust and help them feel more comfortable during the interview session. In Iranian culture, it is customary to present a small gift, like flowers or sweets, when visiting someone's home or meeting them for the first time. This gesture served as the sole incentive for the participants. For data collection, in‐depth and semi‐structured interviews were used. The interview guide is shown in Table 2. All interviews were conducted face‐to‐face. The interviews lasted between 40 and 90 min. The interviews were recorded using a digital voice recorder.

Field notes were taken during the study, documenting participants' nonverbal reactions and emotions that could not be captured with a voice recorder. After the data were coded and analyzed, no new information was added to the existing data, and the sampling concluded with 11 family members. Sampling and data analysis were conducted simultaneously from December 2023 to May 2024.

### 2.4 Data Analysis

The interviews were analyzed via the methods of Graneheim and Lundman (2020) [27]. Themes were not identified in advance; they emerged from the data. First, the researcher listened to each interview several times and then transcribed it manually. The entire text was read thoroughly to gain a general understanding. Each interview was considered an analysis unit and then divided into numerous semantic units. Each semantic unit was also summarized and entered into Maxqdata 2020 for coding. Initial coding was conducted by the second and third authors. Similar codes were grouped, forming subcategories. Similar subcategories then led to the formation of categories. The complete transcripts were coded independently and subsequently combined to verify their similarities. Table 3 lists all the subcategories and categories obtained.

**Table 2:** Categories extracted from the qualitative content analysis.

Some examples of the guiding questions
Who brought the diagnosis of brain death up and how?
What was your and your family's reaction to the brain death diagnosis?
Who suggested organ donation, and how was that brought up?
What was your family's reaction to that suggestion?
What factors encouraged you to consider organ donation?
What factors lead to reluctance and doubts about organ donation?
Please describe your feelings and experiences.
**Some examples of probing questions**
Please elaborate on your experiences.
Can you give an example?

**Table 3:** Interview guide.

Sub subcategory	Sub category	Primary category	Main category
Receiving explanations for brain death in an appropriate manner (*n* = 19)	Discussing donation considerably (*n* = 28)	Organ donation motivators (168)	Difficult dilemma (*n* = 241)
Having enough time to make a decision (*n* = 9)			
Patient's altruism (*n* = 15)	Patient's wishes awareness (*n* = 26)		
Patient's will (*n* = 11)			
Having the support of the health care providers (*n* = 14)	Emotional/psychological support (*n* = 37)		
Having family support (*n* = 23)			
Adequate family knowledge about brain death and organ donation (*n* = 12)	Considering donation reasonable (*n* = 23)		
Understanding the cause of brain death for the family (*n* = 11)			
Positive personal attitude (*n* = 18)	Considering donation valuable (*n* = 54)		
Positive attitude due to religion influences (*n* = 23)			
Positive attitude due to social influences (*n* = 13)			
Receiving explanations for brain death in an inappropriate manner (*n* = 14)	Discussing donation inconsiderately (*n* = 25)	Organ donation demotivators (73)	
Lack of sufficient time to make a decision (*n* = 11)			
Deficiency in family knowledge about brain death and organ donation (*n* = 10)	Distrust in providers (*n* = 29)		
Providers' inadequate knowledge and communication skills (*n* = 11)			
The incomprehensibility of the cause of brain death for the family (*n* = 8)			
Negative personal attitude (*n* = 6)	Considering donation unfavorably (*n* = 19)		
Negative attitude due to religious influences (*n* = 8)			
Receiving negative social reaction (*n* = 5)			

### 2.5 Trustworthiness

To ensure the validity and rigor of the data, the criteria set by Guba and Lincoln (1994) were used. These criteria include transferability, confirmability, dependability, and credibility. To enhance credibility, a period of 6 months was allocated for collecting interviews and analyzing the data. The researchers aimed to facilitate the collection of authentic data by building trust with participants and establishing close communication with them. The researcher made every effort to collect data with maximum diversity in terms of variables such as age, gender, and relationship with the patient. The research team included individuals experienced in various qualitative methods. To ensure the accuracy of the information, transcripts of the interviews were shared with the participants. To clarify ambiguous parts and reach a common understanding of the participants' experiences, some of the findings were shared with them, and our results were confirmed by them. To increase dependability, the texts of several interviews, codes, and extracted categories were given to a researcher familiar with qualitative analysis who was not involved in the study. They were asked to verify the accuracy of the data coding process. To enhance confirmability, efforts were made to accurately record and report the research processes and decisions taken throughout the study to enable others to potentially follow up on the research. To increase the transferability of the research findings, the results were presented to several family members of brain‐dead patients who were not included as participants in the study. Their feedback was solicited regarding the extent to which the findings resonated with their own experiences.

### 2.6 Ethical Consideration

This study, identified by tracking code 98000724, was performed after ethics approval was received under code number IR.KMU.AH.REC.1400.299 from the Ethics Committee of Kerman University of Medical Sciences. All participants provided written informed consent. The authors were affiliated with only one medical center, while the families of the brain‐dead patients interviewed came from various other facilities. Additionally, families were approached for participation at least after 3 months had passed since the organ donation process. It was also clear during the interviews that the interviewers had no prior familiarity with any of the patients' families. Participants were informed that the research was part of the second author's thesis. Owing to the psychological weight associated with reflecting on experiences related to the death of a loved one, complete explanations regarding the study, its purpose, and the confidentiality of the information were provided to the participants. Participants were informed that they could access in‐person or online counseling with a psychologist immediately following the interview session, should they require support. They were informed that they could choose to skip answering some of the questions. Before each interview, explanations were given about recording the participants' voices. The participants were assured that at any point they wanted, the recording could be turned off, and their responses would be noted. It was also assured that the audio file would be deleted immediately after it was transcribed. The participants were assured that they could withdraw from the study at any time, including before the interview, during the interview, or even after the interview and data analysis (e.g., by omitting their participant codes and quotes).

## 3 Findings

### 3.1 Background Information

A total of eleven participants took part in this study, including eight women and three men, with ages spanning from 30 to 60 years. Among them, there were three mothers, two fathers, three spouses, two sisters, and one brother of the deceased individuals. The patients themselves were between 11 and 48 years old, with three being female and eight male. Notably, the majority (seven) experienced brain death as a result of head trauma.

A total of 241 codes, 19 sub subcategories, 8 subcategories, 2 primary categories, and one main category were obtained. The frequencies for each category and subcategory is provided in Table 3. The main category identified was “Difficult dilemma,” which included two categories: “Organ donation motivators” and “ Organ donation demotivators”

### 3.2 Difficult Dilemma

The interviews revealed that the decision‐making process regarding organ donation was very challenging for the participants. They were still in shock from the sudden death of their loved one and were forced to decide to donate their organs. Cultural, social, and religious values, as well as the performance of healthcare staff, sometimes convinced and inclined them to donate organs, while at other times, they caused doubt and hesitation about organ donation.

#### 3.2.1 Organ Donation Motivators

The primary category “Organ donation motivators” included five subcategories: “Discussing donation considerably,” “Patient's wishes awareness”, “Emotional/psychological support,” “Considering donation reasonable,” and “Considering donation valuable.”

##### 3.2.1.1 Discussing Donation Considerably

This subcategory included two sub‐subcategories: “Receiving explanations for brain death in an appropriate manner” and “Having enough time to make a decision”.

###### 3.2.1.1.1 Receiving Explanations for Brain Death in an Appropriate Manner

Nine participants mentioned that providing accurate scientific explanations, as well as empathy toward them, had a significant effect on their willingness to accept brain death and consent to organ donation.

Using clear and simple language without technical terms helped healthcare providers show that they understood the family's feelings and emotional state. This approach made families feel that the medical staff were not just trying to obtain consent from them but were truly supportive during difficult times.

One of the family members mentioned:

… The treatment team was very nice, and they talked to us thoroughly and clarified everything… I asked the doctor about my wife's condition, and he said she was brain‐dead. I asked what that means, and the doctor said it means she's currently alive with machines. I was by her side for an hour, and she did not react. If we disconnect the machines, she will not survive… When we were informed and understood there was no hope and my wife wouldn't come back to life, we decided to donate her organs

(P10, spouse)

###### 3.2.1.1.2 Having Enough Time to Make a Decision

The participants noted the positive impact of having enough time to decide on organ donation and not being forced into a quick decision. This significantly helped patients accept the patient's brain death and agree to donate organs. In this situation, families had enough time to consult with trusted individuals and other knowledgeable doctors in this field, allowing them to make decisions with greater confidence and peace of mind.

One of the participants said, *“We asked the physician to give us the CT scan results of our son so that we could consult with other doctors as well. They provided it to us. We talked to doctors in other cities and sent them the CT scan results, and they said, “No, this is the same as brain death.” (P11, mother)*.

##### 3.2.1.2 Patient's Wishes Awareness

This subcategory includes two subcategories: “patient's altruism” and “patient's will.”

###### 3.2.1.2.1 Patient Altruism

According to the interviews, one of the important factors influencing families' willingness to donate organs was the presence of positive personality and behavioral traits in the patient, such as altruism and selflessness. In this regard, two family members mentioned, *“She was a very faithful and religious girl. She was very kind and self‐sacrificing. She was always thinking about helping others and had a spirit of selflessness and goodwill.” (P3, father) “If my daughter were alive, she would do the same thing. I also gave my consent.” (P8, father).*

###### 3.2.1.2.2 The Patient's Will

The patient's expression of the desire to donate organs to family members during their lifetime had a significant effect on the family's decision to agree to organ donation. From this perspective, one of the families of a patient said, *“My husband had stipulated that if I had an accident or suffered a brain injury, please donate my organs, and well, I followed his wishes.” (P6, wife).*

##### 3.2.1.3 Emotional/Psychological Support

This subcategory includes two subcategories: “Having the support of health care providers” and “Having family support.”

###### 3.2.1.3.1 Having the Support of Health Care Providers

According to the interviews, the active and effective presence of health care providers in empathizing with families, especially those opposed to organ donation, was very important. Seeking help from psychologists and social workers for psychological support for families in this matter has been effective. One participant mentioned this: *“If the person bringing up this issue can empathize with the family and build a good connection with them before mentioning organ donation, that's great. For instance, having a psychologist present is very helpful… My mom was initially against organ donation, but two members of the organ donation team came to our home, talked to her, and she eventually agreed”. (P9, sister).*

According to the participants' experiences, since all family members—not just the decision maker—play a role in the final decision, receiving support from all of them is important. Additionally, some participants described the ongoing emotional and psychological support they received after the organ donation process as a positive experience. This support involved attending the deceased's funeral and socializing with the organ recipient. One participant stated, *“The funeral was very grand; everyone came and said, ‘God bless you,’ and that we had done a good thing.” (P4, mother)*.

###### 3.2.1.3.2 Having Family Support

According to the interviews, some families had relatives who not only were trusted members of the family but also had good communication skills. These relatives, acting as intermediaries between health care providers and the family, played a significant role in securing the family's satisfaction. For example, one participant mentioned, *“My daughter's uncle had spoken with her doctor and others. They said she had brain death and wouldn't come back. He came and told us, discussing organ donation. We completely trusted him, and once he said so, we agreed to sign the paper…” (P3, father)*.

Families that had a trusted relative who was a doctor or had a positive view of organ donation tended to accept organ donation more quickly. For example, one participant mentioned, *“My cousin helped me a lot on this journey to accept this topic more easily and talked to me a lot, and I'm indebted to her” (P6, spouse)*.

##### 3.2.1.4 Considering Donation Reasonable

This subcategory includes two further subcategories: “Adequate family knowledge about brain death and organ donation” and “Understanding the cause of brain death for the family.”

###### 3.2.1.4.1 Adequate Family Knowledge about Brain Death and Organ Donation

Having adequate knowledge about brain death and its irreversible nature significantly influences families' acceptance of organ donation. One of the participants said, *“I participated in Red Crescent first aid classes at the age of 16, and that is how I became familiar with organ donation… I explained that it was brain death, and it is different from being in a coma, and there's no hope for recovery…” (P9, sister)*.

The presence of trusted companions who were familiar with brain death and organ donation topics helped educate the family. One participant mentioned, *“Our cousin is a doctor, and he played a supportive role in this process. After speaking with the physician, he informed us about our brother's condition and said there was no hope for his return to life” (P1, brother)*. The participants also noted their familiarity with organ donation through prior experiences of brain death in their loved ones. Another source of information and awareness about brain death and organ donation was the media. According to the interviews, families' awareness of the vital need for other people for transplant organs reduced the psychological burden of deciding to donate organs, and it inclined them even more toward organ donation. One participant said, *“I had heard before from people around me saying someone had donated organs, and I thought it was a good thing, saving several lives, until I found myself in that situation, and to me, it was the best decision” (P2, mother)*.

###### 3.2.1.4.2 Understanding the Cause of Brain Death in the Family

On the basis of the experiences of the participants, having a clear and defined cause of brain death significantly helped families accept the brain death of their loved ones. Conversely, the sudden emergence of pathology in the patient and the unknown cause of a decrease in the level of consciousness, followed by brain death, made families hesitant about organ donation. One participant mentioned:

When the accident happened, it felt like he was thrown out of the car and hit his head. The emergency responders who arrived at the scene said at that moment that he probably had brain death and sent him to the hospital…

(P11, mother)

##### 3.2.1.5 Considering Donation Valuable

This subcategory includes three sub‐subcategories: “Positive personal attitudes” “Positive attitudes due to religious influences” and “Positive attitudes due to social influences”.

###### 3.2.1.5.1 Positive Personal Attitudes

A personal positive attitude is one of the underlying factors that leads families to agree to organ donation. Individuals who viewed organ donation as a noble and valuable act were more inclined toward it.

I think it is a great and good thing that when someone passes away, their organs can save the lives of several others…

(P8, father)

Some participants even described their agreement to donate organs and save others' lives as a source of good feelings in their lives.

I feel like my life has changed a lot after donating organs, and it is turned upside down.

(P7, sister)

###### 3.2.1.5.2 Positive Attitudes due to Religious Influences

Another factor that could facilitate family satisfaction with organ donation is the family's religious background. Some of the religious motivations for donating organs include the belief that the patient's sins will be forgiven, gaining spiritual rewards for the patient, and considering the patient as a martyr. The participants said:

For the person who has passed away, it is counted as a good deed.

(P1, brother)

My child came to me in a dream and said, ‘I'm in paradise.’ For me, my child has become a martyr, and I'm happy that I decided to donate my child's organs.

(P2, mother)

Among the religious motivations for organ donation is the belief in submitting to divine fate. One participant said:

We told our mom to think that God gave you a gift and then took it back.

(P9, sister)

Additionally, the belief of some families that the patient's life continues through organ donation has facilitated their consent. According to their perspective, with the transplant, the memory of the patient will remain alive in the minds of everyone, especially the recipients.

When I visit my sister's grave, I do not say she has died; I say she is alive because she gave life to a few people.

(P7, sister)

Surely, the person who has one of my sister's organs in them, and who has returned to their normal life because of my sister, will always.

(P10, sister)

###### 3.2.1.5.3 Positive Attitudes due to Social Influences

The positive attitudes of relatives and friends and their encouragement to donate organs also had an impact. One participant said:

They said you are doing a great job and they supported us and no one opposed or said anything to stop us from doing it.

(P10, wife)

They told me that if you do this, it will be great and your child will go to heaven

(P2, mother)

#### 3.2.2 Organ Donation Demotivators

The category of obstacles to organ donation included three subcategories: “Discussing donation inconsiderately,” “Distrust in providers” and “Considering donation unfavorably.”

##### 3.2.2.1 Discussing Donation Inconsiderately

This subcategory included two subcategories: “Receiving explanations for brain death in an inappropriate manner” and “Lack of sufficient time to make a decision.”

###### 3.2.2.1.1 Received Explanations for Brain Death in an Inappropriate Manner

The sudden news of brain death and the lack of groundwork regarding brain death and organ donation left families in doubt and uncertainty. Health care providers announced the death of the brain to them abruptly without informing the families step by step about the progress of the illness. In this regard, family members said:

If someone had explained the whole situation to us on that first day, it would have been much better to discuss organ donation

(P4, mother)

After six days, the nurse told my husband to consent for the organs to be donated. My husband was truly shaken and was very upset at first

(P11, mother)

###### 3.2.2.1.2 Lack of Sufficient Time to Make a Decision

Forcing families to make quick decisions by the donation team added extra psychological pressure on them and made decision‐making difficult. Families said:

Well, everything happened suddenly. At first, they told us there was hope and that he would survive, but in the last few days, out of nowhere, they said no, the doctors said he would not survive…

(P10, wife)

They suddenly said there was no hope and talked about donating our brother's organs, and it would have been better if this process had gone more calmly.

(P1, brother)

##### 3.2.2.2 Distrust in Providers

This subcategory includes three subcategories: “Deficiencies in family knowledge about brain death and organ donation,” “Providers' inadequate knowledge and communication skills” and “Incomprehensibility of the cause of brain death for the family.”

###### 3.2.2.2.1 Deficiencies in Family Knowledge About Brain Death and Organ Donation

Previous experiences of diminished consciousness for reasons other than brain death (persistent coma and vegetative state) among relatives made the patient's family hesitant about organ donation. In this regard, family members mentioned the following:

My wife's brother had an accident and was in a coma for a month. However, he got better. That is why all the relatives said he would be okay.

(P5, wife)

*“Not only in our city but throughout Iran, I think the issue of organ donation is not well established, and there's very little information about it.” (P7, sister)*

###### 3.2.2.2.2 Providers' Inadequate Knowledge and Communication Skills

The inadequate knowledge of health care providers regarding the manifestations and clinical course of brain death and giving false hope about the possibility of the patient returning to prebrain death conditions hinders organ donation. This kind of behavior from health care providers has led to psychological shock for the family.

When we first went to the hospital, they said there was no problem and that he would get better.

(P10, wife)

On the first day at the hospital, they said there's nothing wrong, just a spot of blood in his head

(P5, wife)

The inability to build trust, hide the patient's condition from the family, and avoid giving clear and detailed explanations by health care providers created a lack of consent for organ donation. For example, family members mentioned the following:

Mr…. from the treatment and organ donation team came several times to the patient's side and did not introduce himself. Later, he said this father was so hopeful about his son getting better that I couldn't tell him.

(P4, mother)

###### 3.2.2.2.3 The Incomprehensibility of the Cause of Brain Death for the Family

The interviews revealed that in situations where the underlying cause of brain death was not clear and when the family was not beside the patient at the time of the accident, leading to brain death and transfer to the hospital, there was less willingness to consent to organ donation.

His mom called and said he had a truly bad headache and could not go to school. Come quickly so we can take him to the doctor. I rushed home. He was alert and answered our questions, but as soon as we got to the emergency and they admitted him, he passed out and lost consciousness. They said he had gone into a coma. A few days later, they told us he was brain dead… everything happened so fast, and it was just unbelievable and hard to accept.

(P3, father)

##### 3.2.2.3 Considering Donation Unfavorably

This subcategory includes three further subcategories: “Negative personal attitudes,” “Negative attitudes due to religious influences,” and “Receiving negative social reactions.”

###### 3.2.2.3.1 Negative Personal Attitudes

The belief in the necessity of maintaining the integrity of the body during burial and feeling guilty about the patient's physical state after organ donation has been an obstacle to agreeing to organ donation. In this regard, participants mentioned the following:

My dad was totally against it and kept saying, ‘Are you saying I should let someone cut up my kid!?’

(P9, sister)

###### 3.2.2.3.2 Negative Attitudes due to Religious Influences

Although the religious beliefs of patients' families often contribute to their willingness to donate organs, in some cases, they have the opposite effect. In certain instances, the family's religious beliefs prevented them from accepting brain death and consenting to organ donation. One of the main religious barriers is the belief in miracles and the possibility of the patient returning to life. This was especially true when the incident occurred on religious occasions, leading to heightened hopes for a miracle.

My dad and the rest of the relatives just could not accept the diagnosis of brain death. Because this happened during the nights of Qadr, everyone was hoping for a miracle and that he would come back.

(P7, sister)

###### 3.2.2.3.3 Receiving Negative Social Reactions

The negative attitudes and inappropriate reactions of some acquaintances, stemming from their lack of information, put pressure on the families of patients. One family mentioned:

A lot of people talked behind my back, saying that I teamed up with my dad and sold my sister's organs, and those were painful comments.

(P7, sister)

## 4 Discussion

In this study, the motivators and demotivators affecting the consent of the families of patients with brain death for organ donation were extracted through qualitative interviews. These factors were then compared with those of other studies and discussed. The results concerning “Organ donation motivators” and “Organ donation demotivators” are not addressed separately in the following section. Instead, the motivating and demotivating factors for each topic are discussed and interpreted consecutively.

Several participants in this study noted that providing precise scientific explanations by healthcare providers, combined with understanding the family's situation and showing empathy, significantly impacted their willingness to consent to organ donation. Yousefi et al. [6] also reported that one of the obstacles to organ donation was the inaccurate information provided to families by healthcare providers. Abbasi et al. [12] reported that the inappropriate method or timing of the request by healthcare providers could cause distress among family members and lead to refusal of organ donation. The participants in the study by Ahmadian et al. [5] indicated that receiving bad news suddenly and being asked for consent in inappropriate situations acted as a barrier to organ donation for them. Bjelland and Jones mentioned that improving hospital staff communication, which includes avoiding the use of medical jargon, providing sufficient audio–visual explanations, and understanding that families are trying to cope with a disaster, significantly enhances the process.

The results of the present study showed that pressuring families to make quick decisions by the donation team added extra mental and emotional stress to them, making it harder for them to decide. Abbasi et al. [12] noted that the limited time for decision‐making made the process even more difficult. Ahmadian et al. [5] indicated that the time limitations for decision‐making put them under pressure. Vincent et al. [10] stated that providing enough time for decision‐making and avoiding forcing families into hasty choices has been a vital support aspect for families.

Facilitating experience transfer sessions can improve staff communication about brain death with families. During these sessions, team members as well as family members can share successful interactions, which can positively influence attitudes. By integrating these insights and reviewing existing literature, a protocol can be developed to guide the information shared with families, including optimal communication strategies and timing.

According to the results of the present study, the patient's expressed willingness to donate organs while alive had a significant effect on the family's decision to consent to organ donation. In cases where the patient had not clearly willed to donate organs, the family interpreted personality traits such as kindness and selflessness as the patient's predisposition to donate. Yousefi et al. [6] concluded that families' awareness of their patients' views regarding organ donation facilitated the process. Kerstis et al. [28] mentioned that if the deceased did not have a will, families would focus on the patient's humane personality traits, such as kindness and generosity. Strategies must be implemented within society to promote open discussions regarding individuals' willingness to donate their organs in cases of brain death. These strategies should be culturally appropriate, as perceptions of death, attitudes towards it, and openness to discussing such matters are influenced by cultural factors [7]. Additional research in this domain would prove beneficial.

The results of the present study indicated that empathy and compassion from healthcare workers toward families, especially those opposed to organ donation, were very effective. Seeking help from psychologists and social workers for the psychological support of families influenced their willingness to donate organs. Kerstis et al. [28] reported that the families studied described healthcare providers as supportive, compassionate, and empathetic, which gave them confidence and a sense of satisfaction. Conversely, Abbasi et al. [12] reported that the majority of participants noted that the failure to provide consulting services and the lack of empathy from healthcare workers were identified as major reasons for the refusal to donate organs. Ahmadian et al. [5] reported that the unempathetic behaviors of healthcare providers led to the unmet emotional and supportive needs of family members. On the basis of the participants' experiences, having supportive relatives with a positive attitude toward organ donation played a significant role in gaining the family's consent. Yousefi et al. [6] noted that friends and relatives of participants created a calming environment during times of crisis and provided psychological support during decision‐making, which had a positive and effective impact. On the other hand, the disagreement of some relatives and friends placed the family in confusion, emotional breakdown, and conflicting opinions.

Comprehensive qualitative interviews should be carried out to gain clarity and offer feedback to staff regarding their experiences in caring for brain‐death patients, as well as the factors that affect the development and loss of empathy. Interventions aimed at enhancing empathy and compassion can be implemented for staff. It is also important to provide counseling service to families.

The participants in this study highlighted that having supportive relatives who had a positive view of organ donation greatly improved family consent. Yousefi et al. [6] reported that friends and family members helped create a calming environment during difficult times and offered important emotional support during the decision‐making process. In contrast, disagreements among some relatives and friends resulted in confusion, increased stress, and conflicting opinions within the family. This shows how important social support is for families facing the challenges of organ donation.

According to the interviews, one of the important factors influencing the family's acceptance and welcome of organ donation was having enough knowledge about the conditions of brain death and its irreversibility. In contrast, a lack of sufficient understanding of brain death led to doubt and hesitation among the participants. Kerstis et al. [28] reported that families struggled to understand the concept of brain death. Vincent and colleagues noted that understanding and accepting brain death as death was challenging for families. Ahmadian et al. [5] reported that despite knowing about brain death, family members do not accept the certainty of death. Death was unfamiliar and abnormal for them, which made acceptance difficult. In Kerstis et al.'s [28] study, other participants expressed that they understood the information; however, when they saw their loved one warm and normal, they doubted the diagnosis of brain death. For this reason, they hoped for recovery. Yousefi et al. [6] reported that families hoped their patients would heal and return to them. Abbasi et al. [12] reported that many families had incomplete and even incorrect information about brain death and organ donation, which made them reluctant to donate. A qualitative study by Sarti et al. in Canada found that families of deceased organ donors believe early education on the differences between brain death and coma could improve the donation process [8]. Lalegani et al. [13] reported that familiarity with similar experiences in others and obtaining information through media and social networks facilitated the acceptance of brain death.

The findings stress the importance of strengthening education for families about brain death. More information should be shared through media, and the treatment team should provide ample time for explanations and be open to questions. Educational resources, including videos and software featuring testimonials from families who consented to organ donation, are also recommended. In a qualitative study in Canada by Sarti et al [8]. families of deceased organ donors indicated that having access to individuals who have had similar experiences could improve the organ donation process.

The findings from the present study indicate that participants' understanding of the cause of brain death plays a crucial role in their acceptance of the diagnosis. In contrast, cases involving abrupt pathological changes and an indeterminate cause for the loss of consciousness and subsequent brain death led to increased hesitation among families regarding organ donation. Kerstis et al. [28] reported that when families were informed of brain death and confronted with a request for organ donation, they often experienced a state of shock, which impeded their capacity to process the information provided. Similarly, Yousefi et al. [6] described brain death as an unexpected crisis that left families in disbelief and disorientation. Additionally, Ahmadian et al. [5] noted that family members frequently experienced profound shock and anxiety following the diagnosis of brain death. Abbasi et al. [12] also highlighted that such events typically transpire suddenly, profoundly impacting family dynamics. Consequently, the emotional turmoil experienced makes it challenging for families to fully grasp the situation and make informed decisions regarding organ donation.

On the basis of the experiences of the participants in the current study, the inability to build trust, hiding the patient's condition from the family, giving false hope to families, and avoiding providing detailed and clear explanations by health care providers have contributed to unconsent organ donation. Ahmadian et al. [5] reported that the inadequate and incorrect information provided to participants during the organ donation process upset them. This information is related to the patient's health status, treatment progress, meaning of brain death, certainty of brain death, and donation process. In the study by Kerstis et al. [28], some families mentioned that health staff did not clearly explain the patient's condition to them, leading to distrust and confusion. Direct and honest communication without false hope was appreciated by the participants. Vincent et al. [10] reported that regular updates to families about the patient's condition and disease progression prepare them for potential adverse outcomes and reduce shock. Vincent et al. [10] reported that clarifying the concept of brain death, both ethically and legally, can increase the consent rate and strengthen informed decision‐making. Abbasi et al. [12] noted that a lack of trust in the medical team made the decision‐making process more difficult. Yousefi et al. [6] indicated that distrust in the treatment team can stem from the shock, confusion, and disbelief of the family on the one hand, inappropriate interactions with families, and lack of information about the patients' exact conditions on the other hand.

These findings highlight the crucial role of fostering trust. Sharing the experiences of families with staff can help them grasp the repercussions of unprofessional behavior and the impact of eroded trust. Providing training for staff in this area is vital.

The results revealed that individuals who considered organ donation a good and valuable deed were more inclined toward it. Saxena et al., in India, indicated that for some relatives of deceased organ donors, donating an organ allows their loved ones to live on in another person, relives their grief by giving someone in need a second chance of life [9]. According to the study by Al‐Abdulghani et al. [29], some participants viewed organ donation as an honorable act. Kerstis et al. [28] reported that the motivation for organ donation among participants was the prevention of wasting the patient's life. Altruism plays a crucial role in organ donation. In Iranian culture, there's a strong emphasis on values such as altruism, sacrifice, and mutual support [30]. In Islam, saving a human life is considered one of the highest virtues [31]. The Quran states that whoever saves a life has saved humanity. Belief in Islam generally promotes a positive perspective on organ donation; however, the low level of belief and adherence to Islam among some individuals may diminish this positive outlook.

The participants in the current study indicated that the belief in the necessity of preserving the integrity of the deceased's body was a barrier to consenting to organ donation. Ahmadian et al. [5] noted that participants wished to minimize harm to the body to maintain the dignity of the deceased. Al‐Abdulghani et al. [29] reported that respect for the donor's body was important to the participants. According to Abbasi et al. [12], some participants believed that not burying certain body parts of the deceased was disrespectful to them.

On the basis of the participants' experiences, religious incentives for organ donation included the belief in the forgiveness of the deceased's sins, gaining rewards for the deceased, and the continuation of the patient's life through organ donation. In this context, Kerstis et al. [28] reported that altruistic, spiritual, and religious beliefs influence the decision‐making of relatives. Lalegani et al. [13] mentioned that participants believed that the souls of patients whose organs were donated would find peace after death and that God would grant them a high status after death. Yousefi et al. [6] reported that spiritual and religious beliefs and motivations were among the facilitating factors in organ donation. Families expressed that they consented to organ donation for the sake of God and for the peace of the deceased's soul.

The results indicated that, in some cases, families' religious beliefs hindered the acceptance of brain death and consent for organ donation. One of the main religious deterrents was the belief in miracles and the possibility of the patient returning to life. Abbasi et al. [12] mentioned that some participants hoped for a miracle and that their patients would return to life. Muslims believe that all healing comes from God, asserting that no cure is possible without His divine will. Many maintain faith in God's miraculous healing, even when medical professionals deem a case futile [32]. Others considered organ donation against the principles of their religion for various reasons. Ahmadian et al. [5] reported that some participants were concerned about life. Research conducted in non‐Islamic countries has found that the belief that donation hinders reincarnation and rebirth acts as a barrier to organ donation [11]. Saxena et al. indicated that relatives of deceased organ donors in India held the belief that organ donation might interfere with the donor's afterlife as it compromises bodily integrity [9]. The results suggest that it is important to provide families with religious explanations through a respected and recognized religious leader. These insights could be effectively communicated through educational videos. Furthermore, having a religious figure available to families for sharing this information would be beneficial.

According to the results of this study, the positive attitudes of relatives and friends and their encouragement to donate organs significantly impacted family support. In contrast, the negative attitudes and inappropriate reactions of some acquaintances put pressure on the families of patients regarding organ donation. Al‐Abdulghani et al. [29] reported that social influences significantly affect families' decisions. Abbasi et al. [12] noted that most participants were concerned about being blamed or judged by others. Lalegani et al. [13] mentioned that in some families, several influential yet pessimistic individuals with negative views tried to dissuade the family from donating organs. The findings of this study indicated that organ donation could lead to blame and negative perceptions from friends and relatives toward the family. Yousefi et al. [6] reported that participants were always worried about public opinion because some relatives and individuals thought that the families had sold their patients' organs or had financial expectations from the recipient's family. Claims regarding families being paid for organ donation were also noted in a study conducted in Taiwan [11]. Ahmadian et al. [5] reported that families were concerned about others judging their decisions and about accusations and blame for selling organs and hypocrisy during the decision‐making process. These issues compelled some participants to keep their decisions secret from others. Although under Iranian law and the views of most Iranian clergy, organ donation after death is permitted solely as an altruistic act, while the sale of organs is strictly forbidden [33].

These findings highlight the importance of public education on the topic of brain death. While healthcare staff provide direct education to family members, a lack of understanding among the general public can lead to negative consequences.

## 5 Conclusion

According to the results of this study, cultural, social, and religious factors, as well as the performance of health care providers, play a significant role in families' consent to organ donation. Therefore, to increase families' consent to organ donation, educational and management strategies should be adopted to improve professional communication between health care providers and families. To gain the trust of families, it is recommended that health care providers inform the family of the patient's clinical condition step by step, refrain from giving false hope to the family, and explain the organ donation process to the family in a clear manner. The media can play a very influential role. The media can increase public awareness about brain death and the necessity and conditions of organ donation. The media can influence individuals' attitudes as well as cultural and social issues related to organ donation. It is essential to provide opportunities for more active participation by religious scholars and clergy to answer religious questions and resolve ambiguities regarding brain death. Further studies on organ donation in brain death patients would be useful.

## Author Contributions

**Mehdi Ahamdinejad:** conceptualization; supervision; formal analysis. **Faezeh Ghasemi:** conceptualization; formal analysis; project administration; investigation. **Farideh Razban:** conceptualization; formal analysis; writing – original draft.

## Funding

The authors have nothing to report.

## Disclosure

The corresponding author confirms that this manuscript provides a truthful, precise, and transparent representation of the reported study, and that no significant elements of the research have been excluded.

## Conflicts of Interest

The authors declare no conflicts of interest.

## Data Availability

The data that support the findings of this study are available from the corresponding author upon reasonable request. The qualitative interview analysis is available upon request from the corresponding author. Access will be provided solely for research purposes.
